# 边缘区淋巴瘤诊断与治疗中国指南（2025年版）

**DOI:** 10.3760/cma.j.cn121090-20250612-00273

**Published:** 2025-10

**Authors:** 中国抗癌协会血液肿瘤专业委员会 中华医学会血液学分会淋巴细胞疾病学组 中国临床肿瘤学会（CSCO）淋巴瘤专家委员会 中国惰性淋巴瘤协作组

## Abstract

边缘区淋巴瘤（MZL）是一类起源于淋巴滤泡组织边缘区B细胞的惰性淋巴瘤，占非霍奇金淋巴瘤的5％～15％。MZL具有诊断疑难及治疗选择多样的特点，是淋巴瘤诊治中较为困难的亚型。近年来，随着淋巴瘤整体研究的快速进展，国内外对MZL的认识逐渐提高，为进一步规范我国MZL的诊断和治疗，中国惰性淋巴瘤协作组等组织全国相关领域专家进行讨论，最终形成本指南，供广大医务工作者参考。

## Abstract (en)

Marginal zone lymphoma（MZL）is an indolent B-cell lymphoma arising from marginal zone B cells within lymphoid follicles, accounting for approximately 5％–15％ of non-Hodgkin lymphomas. MZL is characterized by diagnostic challenges and diverse treatment options, making it a challenging subtype in clinical practice. In recent years, with the rapid progress in lymphoma research, the understanding of MZL has improved significantly worldwide. To further standardize the diagnosis and treatment of MZL in China, the Chinese Workshop of Indolent Lymphomas convened a panel of national experts. Based on thorough discussion and consensus, this guideline was developed to provide a reference for medical practitioners.

边缘区淋巴瘤（MZL）是一类起源于淋巴滤泡组织边缘区B细胞的惰性淋巴瘤，占非霍奇金淋巴瘤的5％～15％。MZL兼具起病隐匿、临床表型多样、诊断疑难以及治疗选择多样的特点，诊断常需要依赖多种手段综合判断，是淋巴瘤诊治中较为困难的亚型。近年来，随着淋巴瘤整体研究的快速进展，国内外对MZL的发病机制、预后分层及治疗策略的认识不断加深。为进一步规范我国MZL的诊断和治疗，提高国内MZL的临床管理水平，中国抗癌协会血液肿瘤专委会、中华医学会血液学分会淋巴细胞疾病学组、中国临床肿瘤学会（CSCO）淋巴瘤专委会、中国惰性淋巴瘤协作组组织全国相关领域专家围绕MZL的诊断与治疗策略进行讨论，在广泛达成共识的基础上，最终形成本指南，以期为我国医务工作者在MZL的临床诊疗中提供切实可行的参考。

一、概述

依据2022年更新的第五版世界卫生组织（WHO）分类[1]和临床咨询委员会建立的国际共识分类（ICC）[2]，MZL包括5种亚类：黏膜相关淋巴组织结外边缘区淋巴瘤（MALT淋巴瘤或EMZL）、淋巴结边缘区淋巴瘤（NMZL）和脾边缘区淋巴瘤（SMZL）、原发皮肤边缘区淋巴瘤（PCMZL）和儿童淋巴结边缘区淋巴瘤（PNMZL），其中以EMZL（70％）、NMZL（20％）和SMZL（10％）常见[3]–[5]。边缘区起源的克隆性B淋巴细胞增多（CBL-MZ）是一种可能先于MZL发生的癌前状态[6]。

二、诊断与分型

MZL需要结合形态学、组织病理学、免疫表型、分子遗传学、病原学和发病部位等进行综合诊断（表1）。

**表1:** 不同边缘区淋巴瘤亚型特点[9]–[12]

诊断	原发部位	病原学（发生率，％）	合并症	特殊检查	重现性易位	高频突变基因（突变频率，％）	首选治疗
MALT淋巴瘤	胃	Hp（85）	–	EGD；Hp检查（IHC，或粪便抗原/^14^C-尿素呼气试验）	+3, +18, t（14;18）（q32;q21）IGH/MALT1, t（11;18）（q21;q21）BIRC3/MALT1（15％～50％）, t（1;14）（p22;q32）BCL10/IGH	–	利妥昔单抗或放疗
	眼附属器	鹦鹉热衣原体（0～80）	SS	头颈眼眶MRI；或眼科检查	+3, +18, t（14;18）（q32;q21）IGH/MALT1（15％～20％）, t（3;14）（p14.1;q32）FOXP1/IGH（10％）	TNFAIP3（35）	手术切除或局部放疗
	肺	–	–	支气管镜；或支气管肺泡灌洗；或EGD	+3, +18, t（11;18）（q21;q21）BIRC3/MALT1	–	肺叶切除或利妥昔单抗^a^
	小肠	空肠弯曲菌	–	–	+3, +18, t（11;18）（q21;q21）BIRC3/MALT1, t（1;14）（p22;q32）BCL10/IGH	–	手术切除或利妥昔单抗^a^
	唾液腺	HCV	SS、慢性涎腺炎（70％）	头颈部MRI；耳鼻喉检查；EGD	+3, +18, t（14;18）（q32;q21）IGH/MALT1	TBL1XR1；GPR34（19）	手术切除或利妥昔单抗
	甲状腺	–	桥本甲状腺炎	超声；或甲状腺功能检查	+3, +18, t（14;18）（q32;q21）IGH/MALT1, t（3;14）（p14.1;q32）FOXP1/IGH	CD274（68）； TNFRSF14（53）； TET2（86）	手术切除或利妥昔单抗^a^
	乳腺	–	–	乳腺钼靶X线；或超声；或MRI	–	–	结节切除或利妥昔单抗^a^
NMZL	淋巴结	丙型肝炎病毒	–	外周血或骨髓流式细胞术	+3, +12, +18, 2p和6q扩增	KMT2D（34）；PTPRD（约20）；NOTCH2（20）；KLF2（17）	–
SMZL	脾	丙型肝炎病毒	–	外周血或骨髓流式细胞术	+3, +18, del（7q31–32）（39％）, t（2;7）（p11;q21）IGK/CDK6	KLF2（20~40）；NOTCH2（20）	–
PCMZL	皮肤	伯氏疏螺旋体（欧洲常见）	–	–	+3, +18, t（14;18）（q32;q21）IGH/MALT1, t（3;14）（p14.1;q32）FOXP1/IGH	FAS（>60）	单个病灶优选手术切除，次选放疗；连续病灶优选放疗；多发病灶优选利妥昔单抗
儿童NMZL	淋巴结	–	–	–	+18	–	–
CBL-MZ	外周血	–	–	流式细胞术	–	–	观察随诊

**注** MALT：黏膜相关淋巴组织；Hp：幽门螺杆菌；EGD：食管/胃/十二指肠镜检查；IHC：免疫组织化学染色；SS：干燥综合征；MRI：磁共振成像；HCV：丙型肝炎病毒；NMZL：淋巴结边缘区淋巴瘤；SMZL：脾边缘区淋巴瘤；PCMZL：原发皮肤边缘区淋巴瘤；CBL-MZ：边缘区起源的克隆性B淋巴细胞增多；^a^若已经明确诊断，可优选利妥昔单抗单药治疗，而不行手术切除；–：无内容

（一）细胞形态学特点

小至中等大小淋巴细胞，核染色质致密，胞质较丰富，胞体偏大的中心母细胞和免疫母细胞散在可见，但一般不成片出现，伴有浆样分化的细胞在EMZL和NMZL等亚型中均多见。SMZL细胞形态上可见细胞两极绒毛状突起，与毛细胞白血病不同的是后者通常超过2/3的细胞周边出现绒毛状突起。

（二）组织形态学特点

1. 淋巴结：肿瘤细胞围绕滤泡增生，滤泡间区增宽，挤压滤泡（滤泡植入），但仍可检测到残存的滤泡树突细胞网，这是MZL区别于其他淋巴瘤的重要特征。

2. 骨髓：对于SMZL，骨髓侵犯程度和模式多样，可呈小梁间结节样和窦内浸润，后者对于SMZL具有一定特征性。

3. 脾：对于SMZL，肿瘤细胞同时侵犯脾白髓和红髓，以白髓为主，典型的呈双相性改变，中间为残留的生发中心和套区（深蓝色），周围为胞质丰富、淡染的肿瘤细胞，红髓可见小的肿瘤细胞结节形成，同时侵犯髓窦和髓索。

（三）免疫表型特点

MZL表达全B细胞标志物，如CD19、CD20、PAX5和CD22；通常不表达CD5、CD103；不表达Cyclin D1、SOX11、LEF1和Annexin A1；极少表达生发中心标志物（CD10、BCL6、HGAL和LMO2）。MZL没有特异性免疫标志物，近年来研究显示IRTA1和MNDA是NMZL相对特异的标志物，一般不表达于滤泡性淋巴瘤（FL），可协助MZL与反应性增生以及其他淋巴瘤亚型的鉴别[7]。

（四）遗传学特点

3号和18号染色体三体在所有类型MZL中均较常见，不同MZL类型发生的其他遗传学异常略有不同，详见表1。

（五）病原学特点

慢性病原菌感染和自身免疫性疾病常与MZL相关，如幽门螺杆菌（Hp）感染和胃MALT淋巴瘤相关，鹦鹉热衣原体感染和眼附属器MALT淋巴瘤相关[8]，丙型肝炎病毒（HCV）感染和SMZL相关等（表1）。

基于以上特点，仍有少部分MZL难以诊断，特别是与淋巴浆细胞淋巴瘤/华氏巨球蛋白血症（LPL/WM）、不典型慢性淋巴细胞白血病（CLL）等鉴别，充分的生物学标志物是与其他淋巴瘤亚型鉴别的重要依据，可参照《惰性B细胞淋巴瘤诊断与鉴别诊断中国专家共识（2025年版）》[13]。

三、分期与预后

（一）分期

MZL根据Lugano改良版Ann Arbor系统[14]进行分期（表2），胃肠道MALT淋巴瘤有专门的分期系统[15]（表3）。对于所有类型MZL推荐行颈、胸、腹和盆腔增强CT检查，由于MZL侵犯部位的特殊性，不同部位采取的最优评价方法有所不同。既往研究认为MZL对^18^F-FDG敏感性较FL低，PET-CT仅在考虑转化时进行，但随着PET-CT技术的整体提高，新数据证实大多数MZL为^18^F FDG显影，即将修订的Lugano分类推荐PET-CT作为分期检查手段[16]，因此建议有条件的单位或患者考虑进行PET-CT检查。

**表2:** Lugano改良的分期标准[14]

分期	累及区域
局限期
Ⅰ期	病变累及单一淋巴结引流区（Ⅰ期），或仅局灶累及单一淋巴结外器官或部位（ⅠE期）
Ⅱ期	病变累及横膈同侧的2个或以上的淋巴结引流区（Ⅱ期），或在横膈同侧同时累及1个淋巴结外器官或部位和1个或以上的淋巴结区（Ⅱ_E_期）
Ⅱ期伴大包块	霍奇金淋巴瘤：≥10 cm或大于1/3纵隔；非霍奇金淋巴瘤不同亚型定义不同，6～10 cm均存在，可详细记录最大肿物具体数值
进展期
Ⅲ期	病变累及横膈两侧2个或以上的淋巴结引流区（Ⅲ期），或有脾的累及（Ⅲ_S_期）
Ⅳ期	一个或多个淋巴结外器官或组织的广泛、弥漫性或非连续累及，可伴或不伴有相关淋巴结的累及。骨髓或肝的累及通常认为是Ⅳ期病变
结外（E）	局限的结外侵犯

**表3:** 胃肠道黏膜相关淋巴组织淋巴瘤Lugano分期系统[14]–[15]

Lugano分期		Lugano改良版Ann Arbor分期系统
Ⅰ期	局限于胃肠道（单个或多个非连续性病灶）	ⅠE
Ⅱ期	侵犯到腹腔	ⅡE
	Ⅱ1：局部淋巴结侵犯	ⅡE
	Ⅱ2：远处淋巴结侵犯	ⅡE
ⅡE期	穿透浆膜层侵犯周围脏器或组织	ⅡE
Ⅳ期	播散性结外侵犯或同时侵犯横膈上淋巴结	Ⅳ

（二）预后

对于MALT淋巴瘤，目前常用的预后体系为MALT-IPI。对于SMZL，目前有2个主要的分期系统，分别为意大利淋巴瘤协作组（IIL）积分和简化的HPLL系统[17]–[18]，中国医学科学院血液病医院曾对这2种分期进行检验，发现其并不完全适用于我国SMZL患者，而包括复杂核型（存在3个或3个以上的克隆性染色体数目或结构异常）、LDH水平升高和乙型肝炎病毒（HBV）感染为独立不良预后影响因素的预后系统能更好地区分患者预后[19]。一项前瞻性研究评估了流式细胞术微小残留病（MRD）检测在SMZL中的预后价值，研究证明伴有骨髓侵犯的SMZL患者在接受以抗CD20单抗为基础的治疗后，骨髓MRD的不可检测状态（MRD<0.01％）是独立的有利预后因素[20]。对于NMZL，FL的FLIPI评分也具有一定指导价值。近期研究提出一项涵盖所有MZL的MZL-IPI预后模型，该评分系统基于5个临床因素：LDH升高且大于正常上限值、淋巴细胞绝对计数<1×10⁹/L、HGB<120 g/L、PLT<100×10⁹/L以及NMZL或播散型MZL。根据危险因素数量，患者被分为低危（0个因素）、中危（1～2个因素）和高危（3～5个因素）三组，对应的5年无进展生存（PFS）率分别为85％、66％和37％。该模型不仅可以预测PFS，也具有预测总生存（OS）的能力，值得进一步推广[21]。与其他淋巴瘤亚型一样，疾病早期进展也与较差的预后相关。在Mayo队列中，诊断后前12个月内实现无事件生存（EFS）的患者，其OS期与年龄匹配的正常人群相近，而在12或24个月内发生复发或进展的患者则表现出显著较差的OS[22]。此外，还有其他研究表明诊断时检测到伴有M蛋白以及高Ki-67表达（>20％）与组织学转化风险升高相关，并显著影响PFS[23]–[24]，预后分期系统汇总比较详见表4。

**表4:** 边缘区淋巴瘤预后分期系统汇总

分期内容	MALT-IPI[25]	MZL-IPI[21]	IIL积分[26]	简化HPLL积分[27]
亚型	EMZL	MZL	SMZL	SMZL
因素	年龄>70岁； 分期Ⅲ/Ⅳ； LDH>正常上限	LDH>正常上限； ALC<1×10^9^/L； HGB<120 g/L； PLT<100×10^9^/L； NMZL或播散型MZL	HGB<120 g/L； LDH>正常上限； 白蛋白<35 g/L	HGB<95 g/L； PLT<80×10^9^/L； LDH>正常上限； 脾门外淋巴结肿大
分层	低危（0个因素）； 中危（1个因素）； 高危（2～3个因素）	低危（0个因素）； 中危（1～2个因素）； 高危（3～5个因素）	低危（0个因素）； 中危（1个因素）； 高危（2～3个因素）	A组（0个因素）； B组（1～2个因素）； C组（3～4个因素）
生存	5年EFS率：70％； 5年EFS率：56％； 5年EFS率：29％	5年PFS率：85％； 5年PFS率：66％； 5年PFS率：37％	5年CSS率：88％； 5年CSS率：73％； 5年CSS率：50％	5年LSS率：95％； 5年LSS率：87％； 5年LSS率：68％

**注** MALT-IPI：黏膜相关淋巴组织结外边缘区淋巴瘤国际预后指数；MZL-IPI：边缘区淋巴瘤国际预后指数；IIL：意大利淋巴瘤协作组；HPLL：血红蛋白、血小板、乳酸脱氢酶水平升高，脾门外淋巴结肿大；EMZL：黏膜相关淋巴组织结外边缘区淋巴瘤；MZL：边缘区淋巴瘤；SMZL：脾边缘区淋巴瘤；LDH：乳酸脱氢酶；ALC：淋巴细胞绝对计数；HGB：血红蛋白；PLT：血小板计数；NMZL：淋巴结边缘区淋巴瘤；EFS：无事件生存；PFS：无进展生存；CSS：肿瘤特异性生存；LSS：淋巴瘤特异性生存

四、治疗前评估

（一）必需检查项目

1. 病史和体格检查：特别是自身免疫性疾病史和感染病史，着重注意淋巴结肿大，眼、耳、鼻咽、肝、脾等部位与脏器相关症状和体征。

2. 血液检查

（1）血常规和分类；

（2）外周血流式细胞术免疫分型（EMZL可不做）；

（3）血生化、LDH和β_2_微球蛋白；

（4）免疫球蛋白定量、血清蛋白电泳和免疫固定电泳；

（5）HBV、HCV、人类免疫缺陷病毒（HIV）等感染标志物。

3. 影像学检查：颈、胸、腹和盆腔增强CT。

4. 组织活检

（1）建议淋巴结切除活检，结外组织建议内镜下活检，不推荐行针吸活检。

（2）骨穿活检：NMZL和SMZL必须做，EMZL推荐做，特别是非胃的MALT淋巴瘤。

（3）免疫组织化学染色：至少包括CD20、CD3、CD5、CD10、BCL2、κ/λ、CD21或CD23、Cyclin D1、BCL6。

5. 病原学检查：对于MALT淋巴瘤，建议均进行Hp检查，特别是胃MALT淋巴瘤。其他部位的病原学检查见表1。

（二）可选择的检查项目

1. 血液检查：风湿和自身免疫相关检查；溶血相关检查：Coombs试验、冷凝集素试验。

2. 尿液检查：尿M蛋白。

3. 影像学检查：PET-CT：明确分期、怀疑转化或早期病变考虑进行局部放疗时需考虑进行PET-CT；特殊部位需要考虑MRI或超声检查。心脏彩超（特别是使用蒽环类或蒽醌类药物时），心电图［特别是使用布鲁顿酪氨酸激酶（BTK）抑制剂时］。

4. 遗传学检查：染色体核型（有骨髓侵犯时）、荧光原位杂交和基因突变（表1）。

5. 有条件单位可行超声内镜检查胃肠道部位，并指导内镜下活检。

6. 其他症状体征相关检查，如伴有神经系统表现的患者进行肌电图检查。

五、治疗

证据水平等级参照欧洲肿瘤内科学会（ESMO）指南进行，证据水平分Ⅰ～Ⅴ级，推荐等级分A～E级[28]。

（一）治疗原则

MZL包括的亚型众多，涉及多种脏器，每个亚型都有一些特殊的治疗方式和策略。眼附属器MALT淋巴瘤可参考《眼附属器黏膜相关淋巴组织结外边缘区淋巴瘤诊治中国专家共识（2023版）》[8]。但MZL也有一些共同的治疗原则：①对于有明确病原学感染，首先考虑抗病原微生物治疗，如抗Hp治疗。②对于早期局限性患者，可考虑以下3种治疗方式：手术切除：仅对于部分需要确诊的患者，肿瘤组织整体切除既是诊断方法，也是一种治疗方式，但手术整体不作为常规治疗手段。局部放疗：放疗需要平衡疗效和不良反应两个方面。免疫治疗：通常选用利妥昔单抗单药治疗。③晚期患者需要具备治疗指征后再予以治疗。MZL整体治疗流程见图1。

**图1:**
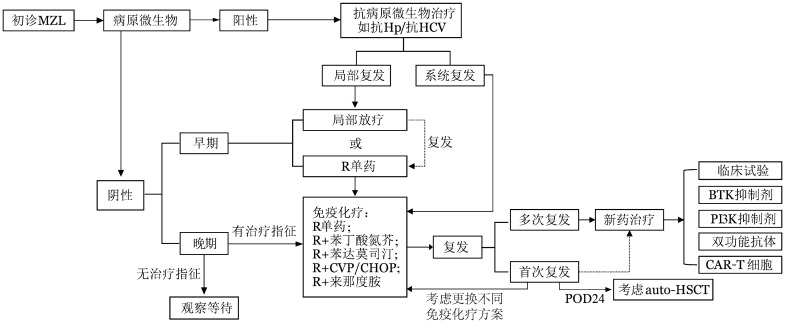
边缘区淋巴瘤（MZL）治疗推荐流程 **注** Hp：幽门螺旋杆菌；HCV：丙型肝炎病毒；R：利妥昔单抗；CVP：环磷酰胺、长春新碱和泼尼松；CHOP：环磷酰胺、多柔比星、长春新碱和泼尼松；POD24：开始治疗后24个月内进展；auto-HSCT：自体造血干细胞移植；BTK：布鲁顿酪氨酸激酶；PI3K：磷脂酰肌醇3激酶；CAR：嵌合抗原受体

（二）抗病原微生物治疗

对于明确相关病原学感染的患者，除疾病进展快等情况外，应先进行抗病原微生物治疗。

1. 抗Hp治疗：对于所有的胃MALT淋巴瘤都可以进行抗Hp治疗【ⅡA】，Hp阴性或伴有t（11;18）易位的胃MALT淋巴瘤疗效可能较差，若抗Hp治疗3～6个月后评估疗效不佳，应进行局部放疗或利妥昔单抗单药治疗。对于眼附属器MALT淋巴瘤，若Hp阳性，抗Hp治疗有效率可达65％[29]，可以尝试【ⅣB】。对于Hp阳性的其他非胃MALT淋巴瘤，抗Hp治疗存在争议，但仍可以尝试。抗Hp治疗方案采取当地推荐的方案进行，一般为三联用药：接受质子泵抑制剂（PPI）治疗4周，并联合2种抗生素，如克拉霉素联合阿莫西林或甲硝唑10～14 d。

2. 抗鹦鹉热衣原体治疗：对于鹦鹉热衣原体阳性的眼附属器MALT淋巴瘤，推荐进行多西环素治疗3周【ⅣB】[30]。

3. 抗伯氏疏螺旋体治疗：伯氏疏螺旋体主要发生在PCMZL，个别报道头孢曲松钠治疗有效率为40％【ⅤC】[31]。

4. 抗HCV治疗：抗HCV治疗HCV阳性的SMZL疗效确切【ⅣB】，以α干扰素（IFN-α）为基础的治疗可实现持续病毒学应答。抗病毒药（雷迪帕韦/索非布韦/利巴韦林/维帕他韦）也可有效清除HCV[32]–[34]。由于抗HCV起效较慢（3～6个月），不建议将其作为需快速控制疾病患者的首选方案。

（三）早期局限型患者

1. 放疗：Ⅰ～Ⅱ期MZL患者，局部病灶受累部位放疗（ISRT）24～30 Gy【Ⅱ～Ⅳ期，A～B】，具体剂量依据不同部位调整。ISRT 24 Gy是首选方案，可使90％或以上的患者实现长期缓解[12]。对于不能耐受标准剂量放疗者，低剂量：4 Gy分2次放疗可使83％患者达到完全缓解（CR）；或12 Gy分4次放疗，CR率达95％，为EMZL可选方案【ⅢB】[35]–[36]。是否适合放疗需要考虑不良反应，胃、皮肤、眼附属器部位可考虑放疗，腮腺、甲状腺、肺部病变放疗非首选，小肠、肾和乳腺需要慎重放疗（表1）[37]。

2. 手术：根治性手术不作为常规治疗手段。但部分患者是通过手术切除而诊断MZL，若无其他病灶，且切缘阴性，可观察随诊，若切缘阳性，可考虑辅助局部放疗或利妥昔单抗单药治疗。

3. 利妥昔单抗单药治疗【ⅢB】：一般为375 mg/m^2^，每周1次，连用4周，不作维持治疗。利妥昔单抗单药治疗尤其适用于病原微生物阴性或抗病毒治疗失败，且不适合接受放疗的MZL患者[37]，对于早期但高肿瘤负荷的患者可考虑联合细胞毒性药物治疗。

（四）晚期患者治疗

1. 治疗指征：晚期MZL患者由于其不可治愈性，需具有治疗指征才能进行治疗。不同MZL治疗指征略有差别，评估主要依据如下原则：受累器官功能异常；症状性脾大或淋巴结肿大或结外肿物；淋巴瘤相关的系统性B症状（发热、盗汗、体重减轻）；淋巴瘤相关的血细胞减少（HGB<100 g/L、PLT<80×10^9^/L、ANC<1.0×10^9^/L）；巨大肿块；疾病持续进展；参加合适的临床试验或具有强烈治疗意愿。NMZL的治疗指征参照FL进行[38]。

2. 治疗方案选择：对于有治疗指征的初治MZL，首选利妥昔单抗为基础的联合化疗方案，联合方案选择可考虑BR（苯达莫司汀+利妥昔单抗）方案、R-CVP（利妥昔单抗+环磷酰胺+长春新碱+泼尼松）方案、苯丁酸氮芥+利妥昔单抗、R2（来那度胺+利妥昔单抗）或R-CHOP（利妥昔单抗+环磷酰胺+多柔比星+长春新碱+泼尼松）方案。各个方案缺乏直接比较。Stil2003[39]和BRIGHT[40]研究表明，BR方案略优于R-CHOP/CVP方案【ⅡA】。R-CHOP方案治疗EMZL和SMZL数据较少。利妥昔单抗维持治疗在MZL中存在争议[41]。

（五）复发难治患者治疗

1. 局部复发：前期经抗病原微生物治疗后复发的患者，考虑局部放疗，具有放疗禁忌证的患者，考虑利妥昔单抗治疗。放疗后局部复发的患者，考虑利妥昔单抗单药治疗。

2. 系统复发：建议行抗CD20单抗联合化疗，联合方案可选与之前非交叉方案，见图1。对于免疫化疗联合治疗后24个月内复发（POD24）的患者，建议考虑新药治疗，并考虑行自体造血干细胞移植巩固。异基因造血干细胞移植相关资料较少，仅限于部分难治患者。

3. 新药治疗

（1）BTK抑制剂：目前伊布替尼、泽布替尼、奥布替尼和阿可替尼均可作为含利妥昔单抗二线治疗后复发难治MZL患者的治疗选择【ⅢB】[16]。奥布替尼是我国唯一获批MZL适应证的BTK抑制剂。

（2）磷脂酰肌醇3激酶（PI3K）抑制剂：目前因PI3K抑制剂的不良反应，美国食品药品监督管理局已经撤销了此类药品的适应证。我国自主研发的PI3K抑制剂林普利塞在Ⅰ期临床试验中治疗复发难治MZL患者获得较好疗效，且相关不良反应较国外报道少[42]–[43]，值得未来在MZL中尝试。

（3）新型抗CD20单抗：奥妥珠单抗（人源化抗CD20单抗）较利妥昔单抗联合化疗（苯达莫司汀、CVP或CHOP方案）在MZL中的疗效并无显著优势【ⅡB】[44]。

（4）细胞治疗：在接受利妥昔单抗联合化疗二线治疗后复发难治的MZL患者中，可考虑靶向CD19的CAR-T细胞治疗【ⅢB】[45]。

（5）其他新疗法：目前在复发难治或初治MZL中进行的Ⅱ～Ⅲ期临床研究包括：BTK抑制剂联合利妥昔单抗、BCL2抑制剂+利妥昔单抗、mosunetuzumab+来那度胺、BTK抑制剂+R2、新型的PI3K抑制剂、靶向CD19的抗体偶联药物（loncastuximab）和靶向CD19的CAR-T细胞治疗，以及抗CD3×CD20双抗等[31]，值得期待。

六、组织学转化

MZL具有向侵袭性淋巴瘤转化的风险，发生率为4％～8％[46]–[48]，其中SMZL（15.7％）和NMZL（17.8％）发生率较其他亚型更高[47]。临床工作中，复发的MZL均需要考虑转化可能，有研究显示初始治疗未达CR、LDH水平升高（>正常值2倍）、4处或以上淋巴结病灶[49]、多处黏膜组织病灶和表达CD5[50]为发生转化的高危因素。在SMZL中的研究显示复杂核型[51]、TP53异常、TNAIFP3突变、CDKN2A/B缺失、6p获得是发生转化的高危因素[52]。

是否发生转化需要组织病理证实，在PET-CT高代谢部位进行活检阳性率高。MZL转化后病理类型绝大多数为弥漫大B细胞淋巴瘤（DLBCL），多数为非生发中心亚型[46]，部分患者可出现TdT表达，但不能仅凭此诊断为淋巴母细胞淋巴瘤。转化后的治疗同DLBCL，治疗后发生转化患者预后较差，而未治疗发生转化患者整体预后与初治DLBCL类似。EMZL Ⅰ/Ⅱ期（Lugano改良的分期标准）发生转化的患者预后相对较好，Ⅲ/Ⅳ期发生转化患者预后较差[46]。

七、特殊亚型

PNMZL：是一类高发于青少年的NMZL，中位发病年龄为16岁，男性为主，绝大多数局限于头颈部，一般不伴有免疫缺陷和EB病毒感染。治疗上以观察随诊为主，需要治疗者可选局部手术切除、局部放疗、免疫治疗或系统化疗，通常获得治愈[53]。

PCMZL：占原发皮肤B细胞淋巴瘤30％～40％，好发于50～60岁的男性，常见四肢和躯干皮肤，皮肤外侵犯罕见。其发病可能与刺身颜料、疫苗接种慢性刺激有关，在欧洲与伯氏疏螺旋体感染相关。需要排除其他系统性淋巴瘤侵犯皮肤。局限病灶以局部治疗为主，如手术切除、小剂量放疗（4 Gy）、局部抹剂氯倍他索、氮芥、咪喹莫特乳膏或局部冷冻疗法，二线治疗考虑利妥昔单抗单药或IFN-α，通常预后良好[54]。

CBL-MZ：近年来研究发现有一类免疫表型与MZL类似，但仅侵犯外周血和骨髓，而无其他组织器官侵犯的亚型，被定义为CBL-MZ[6]，类似于单克隆性B淋巴细胞增多症（MBL），但没有单克隆淋巴细胞绝对计数的限制，患者一般无淋巴瘤相关症状，仅15％～20％的患者最终发展为临床显著的淋巴瘤，一般为SMZL[28]。

八、疗效评价

MZL的疗效评价总体上遵循2014年Lugano淋巴瘤评价标准[55]，但需要依据不同部位进行适当调整，对于特殊部位，需要参考特定标准，如胃、皮肤、球结膜、脾[56]。其中胃MALT淋巴瘤参照GELA分级标准（表5）[28],[57]，SMZL具有自己的独立疗效评价标准（表6）[28],[58]。

**表5:** 胃黏膜相关淋巴组织结外边缘区淋巴瘤GELA组织学分级标准

疗效	淋巴细胞浸润	淋巴上皮病变	基质改变
完全组织学缓解	固有层无或散在浆细胞和小淋巴样细胞	无	固有层正常或无淋巴细胞浸润和（或）纤维化
有微小残留病	固有层/黏膜肌层和（或）黏膜下层可见淋巴细胞聚集或淋巴结节样结构	无	固有层无淋巴细胞浸润和（或）纤维化
有反应性残留病	固有层内可见密集、弥漫或结节状淋巴细胞浸润，围绕腺体延伸	局灶性或消失	固有层局灶性淋巴细胞浸润消退和（或）纤维化
无变化	密集、弥漫或结节样浸润	存在，也可能消失	无变化

**表6:** 脾边缘区淋巴瘤疗效评价标准

疗效	具体标准
完全缓解	器官肿大消失（脾最大长径<13 cm）； 血细胞水平正常（HGB>120 g/L，PLT>100×10^9^/L，淋巴细胞绝对计数>1.5×10^9^/L）； 外周血中没有克隆性B淋巴细胞； 骨髓免疫组化示没有骨髓受侵； 直接抗人球蛋白试验或PET-CT阴性（若治疗前阳性）
部分缓解	可测量病灶缩小≥50％； 临床表现改善>50％； 血细胞减少改善≥50％； 无新发病灶或症状； 骨髓中淋巴瘤细胞侵犯减少，造血储备增加
无效	疾病改善<10％
复发进展	与最佳反应点相比，可测量病灶增加>50％； 出现新发病灶或症状

MZL疗效评价方式应采取与疾病诊断时相同的检测方式，主要的影像学方法为增强CT和MRI，如诊断时采用PET-CT，也可通过前后PET-CT进行评估。

对于SMZL，由于其广泛的骨髓受累，治疗后骨髓能达到流式细胞术MRD阴性（<0.01％）的部分缓解患者，其生存与达CR患者一致。因此，建议有条件的单位定期进行骨髓流式细胞术MRD监测，以优化患者的疗效评估[20]。

九、随访

治疗结束后前2年每3个月随访1次，随后3年每6个月随访1次，以后每年随访1次。随访内容包括病史、体格检查、血常规和生化，以及特殊部位的检查，如眼部检查、胃肠镜检查。影像学检查可依据患者具体情况决定，如前期有淋巴结或脏器肿大，可每3～6个月进行1次影像学复查。
